# Skeletal Muscle Function in Type 1 Diabetes: A Complementary Outcome for Clinical Care and Research

**DOI:** 10.1002/dmrr.70218

**Published:** 2026-08-19

**Authors:** Antonio García‐Hermoso, Jacinto Muñoz‐Pardeza, Ignacio Hormazábal‐Aguayo, Yasmin Ezzatvar

**Affiliations:** ^1^ Navarrabiomed, University Hospital of Navarra, Public University of Navarra (UPNA), Navarre Health Research Institute (IdiSNA) Pamplona Spain; ^2^ Vicerrectoría de Investigación y Postgrado, Universidad de La Serena La Serena Chile; ^3^ Lifestyle Factors With Impact on Ageing and Overall Health (LAH) Research Group, Department of Nursing University of València Valencia Spain; ^4^ Vicerrectoría de Investigación y Postgrado, Universidad de Los Lagos Osorno Chile

## 1 Introduction

Type 1 diabetes has traditionally been conceptualised as a disorder defined by autoimmune β‐cell destruction, insulin deficiency and chronic dysglycaemia [1]. This framework has shaped clinical management and research priorities for decades, with understandable emphasis on insulin therapy, glucose monitoring, and prevention of acute and chronic complications [1]. Yet a purely glucose‐centred view may be limiting. Type 1 diabetes also affects skeletal muscle, a key tissue for insulin action, metabolic regulation and physical function. Increasing evidence suggests that diabetic myopathy represents an important but underrecognized component of the type 1 diabetes phenotype and a potential therapeutic target [2].

This commentary does not aim to provide a systematic review, nor to claim that skeletal muscle function is an established surrogate endpoint for long‐term diabetes outcomes. Rather, we argue that skeletal muscle strength, physical function and exercise capacity are clinically meaningful, undermeasured outcomes that should complement, not replace, glycaemic metrics in type 1 diabetes care. The novelty of this commentary lies in explicitly positioning these dimensions as formal clinical and research outcomes, rather than simply reiterating the established role of exercise in type 1 diabetes.

## 2 Skeletal Muscle as an Undermeasured Component of the Type 1 Diabetes Phenotype

This measurement gap is clinically relevant. Human studies suggest that people with type 1 diabetes may exhibit structural, metabolic and functional alterations in skeletal muscle, including reduced muscle strength, altered muscle morphology, impaired mitochondrial function, and lower exercise capacity [3, 4, 5, 6]. Among the most informative studies, Dial et al. [3] reported impaired muscle function and altered skeletal muscle morphology across the adult lifespan in people with type 1 diabetes, with findings consistent with accelerated neuromuscular ageing even in the absence of major overt diabetic complications. These observations support the view that skeletal muscle involvement is not merely peripheral but may represent an undermeasured component of the type 1 diabetes phenotype.

The most clinically relevant phenotype is unlikely to be muscle mass alone. Muscle strength, physical function and exercise capacity may better capture what matters clinically: the ability to exercise, tolerate daily activities, maintain independence, and sustain long‐term physical activity. These structural characteristics remain important, but should not be interpreted as sufficient markers of muscle health. The emerging literature on sarcopenia and dynapenia in type 1 diabetes reinforces the need to assess muscle health as a multidimensional construct combining muscle strength, muscle quantity or quality, and physical performance [7].

**FIGURE 1:**
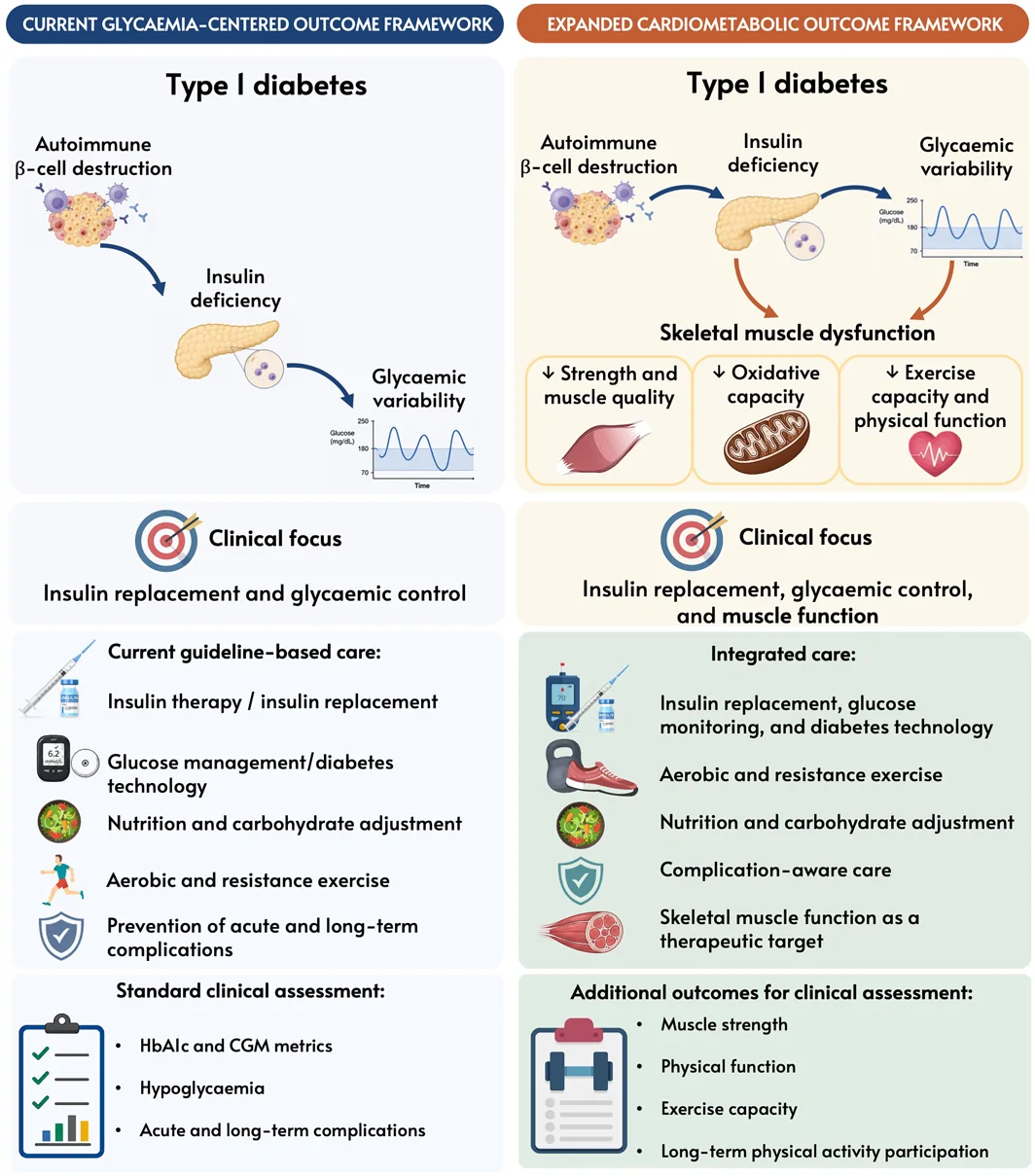
Conceptual expansion from a glycaemia‐centred outcome framework to an expanded cardiometabolic framework in which skeletal muscle strength, physical function, and exercise capacity are incorporated as complementary clinical and therapeutic outcomes in type 1 diabetes.

Figure 1 illustrates the conceptual shift from a traditional glucose‐centred model of type 1 diabetes towards an expanded cardiometabolic model in which skeletal muscle function is considered a clinically relevant outcome.

## 3 Why Muscle Function Matters Beyond Glycaemic Metrics

The clinical relevance of this perspective is substantial. First, skeletal muscle abnormalities in type 1 diabetes are associated with lower exercise capacity, although this relationship is likely bidirectional: dysglycaemia, glycaemic variability, complications and fear of hypoglycaemia may reduce exercise tolerance, while low tolerance may further limit physical activity [4]. Second, because skeletal muscle is the principal site of insulin‐stimulated glucose disposal, impaired muscle health is plausibly relevant to insulin sensitivity and whole‐body glycaemic regulation [8]. Third, persistent impairments in skeletal muscle function and morphology may help explain why some individuals with type 1 diabetes exhibit reduced functional performance that is not fully captured by glycaemic metrics alone [3, 4].

These considerations have practical implications, but they should not be reduced to a simple message that resistance training is absent from current guidelines. Current American Diabetes Association standards already recommend resistance exercise for people with diabetes [1]. The unresolved issue is that skeletal muscle health is rarely treated as a therapeutic outcome. Clinical and research frameworks in type 1 diabetes still prioritise glycaemic metrics, whereas direct assessment of muscle strength, physical function and exercise capacity remains uncommon.

From a pragmatic clinical perspective, skeletal muscle function could be assessed using simple, accessible, and scalable tools. Handgrip dynamometry provides a feasible measure of muscle strength and has identified lower grip strength in adults with type 1 diabetes than in controls [6]. Sit‐to‐stand tests may complement this assessment by capturing lower‐limb function and physical performance. When available, body‐composition assessment may complement functional testing, provided that standardised protocols are used. In selected clinical or research contexts, exercise capacity measures may further characterise functional limitation, particularly given the lower exercise capacity reported in people with type 1 diabetes [4]. These tools should complement glycaemic outcomes by identifying functional vulnerability and monitoring response to intervention. Diabetes technology may improve glucose safety during exercise, but it does not capture changes in strength, physical function or exercise capacity.

## 4 From Resistance Training to Muscle‐Directed Diabetes Care

Resistance training is one practical strategy to target muscle strength and function. Recent randomised trial data in both adults [9] and children and adolescents [10] suggest that resistance training can increase muscle strength and can be implemented without increasing adverse glycaemic events. These findings should not be overinterpreted as proving that resistance‐training‐induced gains in muscle outcomes independently translate into long‐term cardiometabolic benefit, although the broader benefits of regular exercise in diabetes are well established [1]. Skeletal muscle function should therefore be recognised as a legitimate therapeutic outcome, not simply as a proxy for resistance training.

A broader approach is needed. Resistance training should be integrated with aerobic exercise, nutrition, appropriate insulin and carbohydrate adjustment, glycaemic management, diabetes technology, and attention to diabetes‐related complications that may affect neuromuscular function [2]. In this sense, the target is not resistance training itself, but the preservation and improvement of muscle strength, physical function, and exercise capacity across the life course.

## 5 Biological Rationale and Future Therapeutic Targets

Chronic dysglycaemia and non‐physiological peripheral insulin delivery may disrupt skeletal muscle homoeostasis through interacting alterations in anabolic signalling, mitochondrial energetics, proteostasis, inflammation, and regeneration [5, 11]. Emerging molecular pathways may further contribute to these disturbances. Under metabolic stress, cytosolic DNA accumulation may aberrantly activate the cGAS–STING pathway [12], promoting sterile inflammation and cellular damage, whereas dysregulation of the AMPK/SIRT1/PGC‐1α axis may impair mitochondrial biogenesis and oxidative metabolism [13]. Macrophage dysfunction, including failure to transition from pro‐inflammatory to reparative phenotypes, may prolong inflammatory signalling and compromise satellite‐cell activation and muscle repair [14]. Mechanosensing pathways such as Piezo1 may also link mechanical loading with muscle maintenance and regeneration [15]. Although evidence for these pathways specifically in type 1 diabetes remains limited and largely preclinical, they strengthen the biological plausibility of skeletal muscle dysfunction as a modifiable outcome and may eventually inform more individualised exercise or adjunctive therapeutic strategies.

## 6 Discussion

Muscle strength, physical function, and exercise capacity are clinically relevant outcomes because they directly capture the ability to exercise, perform daily activities, maintain independence, and sustain long‐term physical activity. Muscle function should therefore be considered a complementary, patient‐relevant outcome, not a replacement for glycaemic metrics or a validated surrogate endpoint. Future studies should determine whether improvements in these outcomes also influence insulin requirements, continuous glucose monitoring metrics, hypoglycaemia risk, quality of life, and long‐term physical activity participation.

Importantly, the relevance of skeletal muscle assessment is unlikely to be uniform across all people with type 1 diabetes. It may be particularly informative in older adults with long‐standing disease [3, 7], individuals with subclinical or established neuropathy [2], patients with reduced exercise capacity [4], and those with apparently acceptable glycated haemoglobin levels but unfavourable body composition, including low muscle mass, increased adiposity or normal‐weight obesity [7]. Identifying these subgroups may help move the field from a homogeneous view of type 1 diabetes towards a more functionally informed clinical phenotype.

Taken together, these observations support a broader therapeutic model of type 1 diabetes. Glycaemic management remains essential, and resistance training is already recommended. The unresolved issue is whether skeletal muscle strength, physical function and exercise capacity should be measured and treated as clinically relevant cardiometabolic outcomes in type 1 diabetes care. Skeletal muscle lies at the intersection of metabolism, physical function, and daily living in type 1 diabetes. Continuing to treat it as an afterthought may unnecessarily constrain the scope of diabetes care.

## Author Contributions

**Antonio García‐Hermoso:** conceptualization, writing – original draft, writing – review and editing. **Jacinto Muñoz‐Pardeza:** writing – review and editing and intellectual content. **Ignacio Hormazábal‐Aguayo:** writing – review and editing and intellectual content. **Yasmin Ezzatvar:** conceptualization, writing – review and editing, and intellectual content. All authors read and approved the final version of the manuscript and agreed to its submission.

## Funding

The authors have nothing to report.

## Conflicts of Interest

The authors declare no conflicts of interest.

## Data Availability

No datasets were generated or analyzed for this article.
